# Temporal Changes in Mitochondria-Centric Excitotoxic Responses Following Severe Penetrating Traumatic Brain Injury

**DOI:** 10.3390/biomedicines13071520

**Published:** 2025-06-21

**Authors:** Hiren R. Modi, Sudeep Musyaju, Anke H. Scultetus, Jignesh D. Pandya

**Affiliations:** Brain Trauma Neuroprotection Branch, Walter Reed Army Institute of Research (WRAIR), Silver Spring, MD 20910, USA

**Keywords:** severe traumatic brain injury, time course, mitochondrial damage, excitotoxicity, calcium homeostasis, cell death

## Abstract

**Background/Objectives:** Traumatic brain injury (TBI) remains a significant and urgent medical concern for the US military. TBI triggers excitotoxic responses immediately, involving mitochondrial dysfunction characterized by loss of calcium (Ca^2+^) cycling, membrane damage and increased cell death. However, a comprehensive understanding of mitochondria-centric excitotoxic responses over time has yet to be fully demonstrated after severe TBI. The current study evaluated mitochondria-centric time course responses between 30 min and 2 weeks (seven time points) after penetrating TBI (pTBI). **Methods**: Anesthetized adult male Sprague-Dawley rats were subjected to either 10% unilateral pTBI or Sham craniectomy. Animals were euthanized at various time points, and mitochondria were isolated from the injury core. **Results**: Post-injury mitochondrial Ca^2+^ homeostasis was significantly compromised in pTBI compared to the Sham group. In parallel, mitochondrial membrane integrity markers, including cytochrome c (Cyt C) and voltage-dependent anion channel (VDAC), showed significant reduction over time post-pTBI. Apoptosis-responsive markers, such as glyceraldehyde 3-phosphate dehydrogenase (GAPDH) and B-cell lymphoma 2 (Bcl-2), exhibited elevated responses over time post-pTBI. **Conclusions**: Our results demonstrate profound insights into elevated excitotoxic mitochondrial damage after severe TBI. This time course study uncovers novel mitochondrial targets involved in TBI excitotoxicity and offers mitigation opportunities to alleviate excitotoxic responses after penetrating TBI.

## 1. Introduction

Traumatic brain injury (TBI) remains a significant concern among US troops and is often labeled as a “hidden wound of war”. Over the past decade, around 417,000 US service members worldwide were affected by TBI [1]. The majority of TBIs are mild in nature; however, even moderate to severe TBI can lead to post-injury consequences, and survivors often require long-term care for sustained recovery [2]. The biochemical and structural alterations in the brain following TBI have been linked to the early development of neurodegenerative diseases such as Alzheimer’s Disease (AD), Parkinson’s Disease (PD), and Chronic Traumatic Encephalopathy (CTE). Unfortunately, there are no FDA-approved available treatments targeting TBI pathology, highlighting the urgent need to test and validate potential neuroprotective compounds for improved outcome in the military and veteran population.

Several pre-clinical TBI models, including penetrating TBI (pTBI), controlled cortical impact (CCI), blast-induced TBI (bTBI) and closed head injury (CHI), have ascertained that mitochondrial dysfunction is a common indicator of cellular damage [3,4,5,6] and plays a pivotal role in post-injury excitotoxic events. Mitochondria-centric calcium (Ca^2+^) handling, energy metabolism, and redox homeostasis are key cellular mechanisms involved during the acute phase of TBI. Any imbalance in these processes may prompt the activation of downstream cell death pathways, impacting behavioral outcomes following TBI. Multiple published reports have reviewed pre-clinical TBI models and post-injury cellular mechanisms of TBI [7,8,9,10,11], including mitochondrial homeostasis [12,13,14].

During the excitotoxic phase of brain trauma, abnormally elevated excitatory amino acids (EAAs), including glutamate and aspartate, as well as the activation of pre-synaptic receptors and ionic channels like voltage-gated Ca^2+^ ion channels *N*-methyl-d-aspartate (NMDA) and aminomethylphosphonic acid (AMPA), are observed at the site of injury [15,16]. This hyperactivation of ionic channels leads to a deleterious intracellular Ca^2+^ influx. Functional mitochondria regulate intracellular Ca^2+^ levels through mitochondrial membrane potential (ΔΨm). However, excessively high cellular Ca^2+^ levels can overwhelm the mitochondria, potentially leading to excitotoxic cell death after TBI [17].

Post-injury, excitotoxic cell death is further influenced by the release of mitochondrial protein such as cytochrome c (Cyt C), Bcl-2 family, voltage-dependent anion channel (VDAC), caspases, and apoptosis-inducing factor (AIF), all contributing to mitochondria-centric neuronal death after brain trauma [12,18,19,20]. Moreover, early opening of the mitochondrial permeability transition pore (mPTP) disrupts mitochondrial matrix Ca^2+^ levels leading to a loss of ΔΨm and compromises mitochondrial bioenergetics [21]. Intracellular Ca^2+^ also plays a crucial role in maintaining mitochondrial redox homeostasis, acting as a signaling molecule and influencing cellular responses to oxidative stress [22]. An overload of Ca^2+^ in mitochondria correlates with heightened oxidative stress (ROS) and reduced oxidative function [22].

Our published data on the pre-clinical pTBI model have noted a pivotal role of mitochondrial function and cross-talk among various aspects of mitochondrial homeostasis, such as Ca^2+^ dynamics, bioenergetics, antioxidants, redox, and apoptosis/necrosis, following severe TBI [23,24,25,26]. However, the temporal effects of mitochondria-mediated downstream effects remain unexplored after severe TBI. The current study examined the temporal profile of such markers between 30 min and 2 weeks post-injury (seven time points) after pTBI. This study offers valuable insights into new therapeutic targets aimed at modulating Ca^2+^ homeostasis and apoptotic cell death in the aftermath of severe TBI.

## 2. Material and Methods

### 2.1. Reagents

Mitochondrial isolation- and respiration-related reagents were purchased from Sigma (St. Louis, MO, USA) as previously described [23,24,26]. A BCA protein assay quantification kit was purchased from Fisher Scientific (Hampton, NH, USA). Fluorescent dyes, tetramethyl rhodamine ethyl ester (TMRE) and calcium green (CaG5N), were purchased from Fisher Scientific (Waltham, MA, USA). Western blot antibodies were purchased from known vendors.

### 2.2. Animals

Adult male Sprague-Dawley rats (280–350 g, Charles River Laboratories, Raleigh, VA, USA) were used for this study. The animals were housed under a normal 12 h light/dark cycle (lights on at 0600 EST) in a well-ventilated facility accredited by AAALAC international and were allowed 7 days for acclimation to the housing facility before any experimental procedures were performed. All procedures were approved by the Institutional Animal Care and Use Committee (IACUC), Walter Reed Army Institute of Research (WRAIR). Animal studies were conducted in compliance with the Animal Welfare Act, the Guide for the Care and Use of Laboratory Animals (National Research Council), and other federal statutes and regulations relating to animals and experiments involving animals. On the day of the experiment, age-matched animals were randomized into two experimental groups.

### 2.3. Penetrating Traumatic Brain Injury (pTBI)

The pTBI surgical procedure was previously described in detail [26]. Briefly, all surgical procedures were performed under isoflurane anesthesia (3–5% for induction and 2% for maintenance) and aseptic conditions with careful monitoring of vital physiological signs. The pTBI apparatus consists of a specifically designed probe (Kadence Science, Lake Success, NY, USA), a stereotaxic frame (Kopf, Tujunga, CA, USA), and a hydraulic pressure pulse generator (4B080; MITRE, Bedford, MA, USA). The probe was made of a 20G stainless steel tube with fixed perforations along one end, which was sealed by a piece of airtight elastic tubing. The probe was secured on the probe holder with the un-perforated end attached to the pulse generator, angled at 50° from the vertical axis and 25° counterclockwise from the midline. During the surgical procedure, normothermic (~37 °C) core body temperature was maintained using a heating blanket (Harvard Apparatus, South Natick, MA, USA).

Before performing the surgical procedures, animals were randomized into experimental groups. All Sham animals underwent craniectomy but no insertion of the pTBI probe. Under isoflurane anesthesia (2%; in air/oxygen mixture), the animal’s head was secured in the stereotaxic device. After a midline scalp incision, a right frontal cranial window (diameter = 4 mm) was created using a dental drill to expose the right frontal pole (+4.5 mm AP, +2 mm ML to bregma). In pTBI animals, the pTBI probe was then advanced through the cranial window into the right hemisphere to a depth of 1.2 cm from the surface of the brain. Once the probe was in place, the pulse generator was activated by a computer to release a pressure pulse calibrated to produce a rapid expansion of the water-filled elastic tubing to create an elliptical-shaped balloon (diameter = 0.633 mm) to a volume equal to 10% of the total brain volume. This rapid inflation/deflation (duration = 40 ms) produced a temporary cavity in the brain. After deflation, the probe was immediately retracted from the brain, and the skin incision was closed with wound clips. After the completion of the surgical procedures, animals were survived to different post-injury experimental time points (i.e., 30 min, 3 h, 6 h, 24 h, 3 days, 7 days, and 14 days) to measure mitochondrial functions after pTBI. This well-established pTBI model has been evaluated for the histopathological changes [27].

### 2.4. Mitochondrial Isolation

On the terminal day of each experimental time point, both pTBI and Sham animals were humanely euthanized, and their brains were quickly removed to isolate mitochondria using mitochondrial isolation buffer (MIB). From the ipsilateral hemisphere, the frontal cortex (FC) and striatum (ST) regions were dissected and pooled together for mitochondrial isolation. The pooled brain regions represent the injury core (FC + ST), where extensive cell death may occur due to the initial mechanical force (primary injury) and excitotoxicity (secondary injury).

All reagents, centrifuges, and tubes were maintained at 4 °C throughout mitochondrial isolation using the Ficoll-based mitochondrial isolation procedure described previously. Each tissue sample was homogenized in 2 mL of MIB (215 mM mannitol, 75 mM sucrose, 0.1% BSA, 20 mM HEPES, 1 mM EGTA, pH 7.2). Homogenates were centrifuged at 1300× *g* for 3 min, and resultant pellets were discarded to remove the cell debris and nuclei. The supernatants were transferred into new tubes and topped with MIB. Supernatants were centrifuged at 13,000× *g* for 10 min to obtain differential mitochondria (DM). The DM fraction contains crude mitochondria, synaptosomes, myelin fragments, and other organelles, such as ER, lysosome, and small vesicles. The DM fractions were resuspended in 0.5 mL MIB, and then disrupted in a nitrogen cell disruption chamber (model 4639, Parr Instruments, Moline, IL, USA) at 1200 psi for 10 min. Each DM fraction was placed onto a discontinuous Ficoll gradient (1.5 mL 7.5% Ficoll solution layered on top of 1.5 mL 10% Ficoll solution) and centrifuged at 100,000× *g* (Beckman-Coulter Optima XE-90 w/SW-55-Ti Rotor; 32,000 rpm; Beckman Coulter, Inc., Brea, CA, USA) for 30 min. The resulting mitochondrial pellets were carefully separated, re-suspended, washed with MIB without EGTA (MIB^−^), and centrifuged again at 10,000× *g* for 10 min. The resultant ultrapure mitochondrial pellets were re-suspended in MIB^−^ to achieve a final protein concentration (~10 mg/mL). After isolation, mitochondria were immediately used for Ca^2+^ buffering experiments and stored at −80 °C for Western blot analysis. The absolute protein concentration of mitochondrial samples was determined using a BCA protein assay kit.

### 2.5. Mitochondrial Calcium Buffering Capacity

The mitochondrial real-time Ca^2+^ buffering capacity assay was conducted in a spectrofluorometer using 2 mL KCl respiration buffer (125 mM KCl, 2 mM MgCl_2_, 2.5 mM KH_2_PO_4_, 0.1% BSA, 20 mM HEPES at pH 7.2) in a constantly stirred temperature-controlled cuvette at 37 °C. Freshly isolated mitochondrial protein (200 μg) was added in a cuvette along with fluorescent dyes, calcium green^TM^-5N (CaG5N 100 nM; Ex λ, 506 nm; Em λ, 532 nm) to monitor extra-mitochondrial Ca^2+^ and tetramethyl rhodamine (TMRE 150 nM; Ex λ, 550 nm; Em λ, 575 nm) to simultaneously monitor the change in mitochondrial membrane potential (ΔΨm) as described previously [25,28,29,30,31]. The real-time baseline recording of CaG5N and TMRE traces was initiated when mitochondrial protein was co-incubated with flourescent dyes in 2 mL KCl buffer (0–1 min) for each assay. A sequential addition of mitochondrial substrates and stressors was conducted to measure the mitochondrial viability response. First, 5 mM pyruvate and 2.5 mM malate were added at 1 min, followed by 150 μM ADP at 2 min, and then 1 μM oligomycin at 3 min. At 5 min, continuous infusion of Ca^2+^ (32 mM CaCl_2_) was initiated via a Hamilton syringe loaded in a syringe infusion pump (KD Scientific, Holliston, MA, USA) to deliver 80 nmols Ca^2+^/mg protein/min (flow rate: 0.5 μL CaCl_2_/min) for approximately up to 30–40 min, until the mitochondria underwent ex vivo mPTP opening. The CaG5N signal was used to determine mPTP opening, evident from the inability of mitochondria to take up Ca^2+^ from the media [25,28,29,30,31]. The TMRE and CaG5N representative traces reflecting real-time Ca^2+^ cycling and alteration in ΔΨm in the Sham and pTBI cohorts are shown.

### 2.6. Western Blots

At multiple post-injury time points, excitotoxic cell death regulation markers were quantified in mitochondrial samples isolated from the pTBI and Sham groups using the Western blot procedure described previously [25,26,32]. On the day of the experiment, samples were diluted to 1 µg/µL in Milli-Q water, 4x XT Sample Buffer (Bio-Rad, Cat # 1610737, Hercules, CA, USA), and XT reducing agent (Bio-Rad, Cat # 1610737). Samples were then heated to 95 °C for 5 min and loaded onto commercially prepared 4–12% Criterion™ XT Bis-Tris Protein Gel (Bio-Rad, Cat # 3450124). Mitochondria samples were loaded at 10 µL per lane to achieve a total of 10 µg protein. The Chameleon Duo pre-stained protein ladder (LI-COR, Cat # 928-60000, Lincoln, NE, USA) was included on all gels. Following electrophoresis, samples were transferred to a methanol-activated Immobilon FL PVDF membrane (Millipore, Cat # IPFL20200, Bedford, MA, USA) with Trans-Blot^®^ Turbo™ Transfer System (Bio-Rad, Cat # 1704150).

After transfer, PVDF membranes were stained for total protein detection using the REVERT™ kit (LI-COR, Cat # 926-11010) following the manufacturer’s instructions. Membranes were submerged in Revert stain for 10 min, washed twice for 1 min each with wash solution (LI-COR, 6.7% (*v*/*v*) glacial acetic acid, 30% (*v*/*v*) methanol, in water), and imaged immediately at the 700 nm channel of the Odyssey imaging system (LI-COR). The total protein stain was then removed by 15 min incubation in reversal solution (LI-COR, 0.1 M sodium hydroxide and 30% (*v*/*v*) methanol in water) followed by three quick washes in Tris Buffer Saline (TBS, Fishers, Cat # 7447-40-7, Hampton, NH, USA). The membranes were then blocked with Odyssey blocking buffer (LI-COR, Cat # 927-50000) and incubated with primary antibodies prepared in the blocking buffer overnight at 4 °C in the orbital shaker.

The primary antibodies used in the different experiments were Cyt C (1:1000, Cat # ab133504), VDAC (1:1000, Cat # ab15895), GAPDH (1:10000, Cat # ab181602), and Bcl-2 (1:1000, Cat # ab59348), purchased from Abcam (Cambridge, MA, USA). All incubation and washing steps were performed according to the published method [32]. The intensity of the bands was visualized and quantitated using the Odyssey imaging system (LI-COR).

### 2.7. Statistical Analysis

All results were expressed as mean ± standard error of the mean (SEM). For the Ca^2+^ buffering capacity parameter, one-way ANOVA followed by Dunnett’s post hoc test was conducted with respect to the Sham value to determine significant differences across various pTBI time points. For molecular markers, a two-tailed unpaired *t*-test assuming equal variances was used to determine the significance between pTBI vs. Sham group at each time point. To achieve statistical significance, animals in each group were randomized, with a minimum of 6 animals per group. Statistical comparisons were conducted using Prism-GraphPad software (Version 8), and statistical significance was defined as *p*-value * *p* ≤ 0.05.

## 3. Results

### 3.1. Temporal Loss of Calcium Buffering Capacity Post-pTBI

Mitochondrial Ca^2+^ buffering capacity provides a critical understanding of the temporal progression of mitochondria-centric excitotoxic responses following TBI. It plays a significant role in various downstream cellular processes, including bioenergetics failure, elevated oxidative stress, and activation of cell death pathways after TBI. The real-time ex vivo measurement of altered mitochondrial Ca^2+^ buffering capacity and loss of membrane potential are early indicators of mitochondrial impairment and may serve as upstream targets for stabilizing post-injury mitochondrial dysfunction and secondary injury progression after TBI.

**Figure 1:**
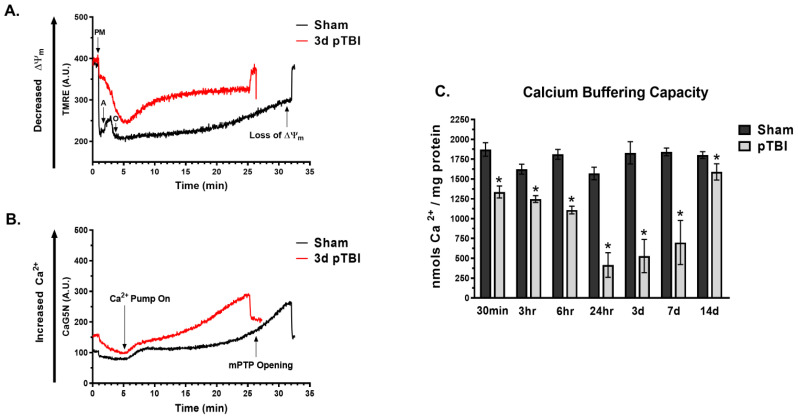
Time course evaluation of mitochondrial Ca^2+^ buffering capacity at the injury core post-pTBI. As illustrated in parallel real time (**A**) TMRE and (**B**) CaG5N traces for both pTBI and Sham cohorts at 3 days post-injury using spectrofluorometer. The addition of pyruvate and malate (PM) at 1 min causes a marked downward deflection due to increased ΔΨm. Following the addition of ADP (A) at 2 min, the loss of ΔΨm is indicated by an upward deflection as ΔΨm is utilized to phosphorylate ADP to ATP via proton flow through the ATP synthase. The addition of the ATP synthase inhibitor, Oligomycin (O), at 3 min results in maximum ΔΨm as proton flow is inhibited. The Ca^2+^ infusion began at 5 min (infusion rate 80 nmol of Ca^2+^/mg protein/min) and monitored by CaG5N fluorescence and was illustrated by the initial upward deflection followed by constant Ca^2+^ uptake into the matrix. The subsequent rise in TMRE and CaG5N fluorescence after Ca^2+^ infusion accompanied by a loss of ΔΨm signifies mitochondrial permeability transition and mPTP over time, and mitochondria undergo cellular stress post-injury. (**C**) This histogram represents quantification of mitochondrial Ca^2+^ buffering capacity (nmols Ca^2+^/mg protein) over time. Ca^2+^ buffering capacity was significantly decreased at 30 min (29% decrease), 3 h (23% decrease), 6 h (39% decrease), 24 h (76% decrease), 3 days (71% decrease), and 7 days (62% decrease) and non-significantly lower at 14 days (12% decrease) compared to the Sham group. Mitochondria sequestered significantly lower amounts of Ca^2+^ in pTBI compared to the respected Sham group (N = 6–9 animals per group, * *p* < 0.05). One-way ANOVA followed by Dunnett’s post hoc test against the Sham values.

We measured the Ca^2+^ buffering capacity from FC + ST brain regional mitochondria isolated from the pTBI and Sham cohorts at various post-injury time points. Realtime mitochondrial Ca^2+^ buffering, ΔΨm, and mPTP opening time were measured at 30 min, 3 h, 6 h, 24 h, 3 days, 7 days, and 14 days post-injury time points (Figure 1). The typical traces of TMRE and CaG5N at 3 days post-injury are illustrated in Figure 1A,B, respectively. The TMRE trace of the Sham group showed a normal pattern of ΔΨm buildup corresponding to the addition of mitochondrial substrates/stressors (e.g., PM, ADP, and Oligo) within 0–5 min intervals. In contrast, pTBI showed an impaired ΔΨm buildup pattern corresponding to the addition of mitochondrial substrates/stressors. At 5 min and onwards, the initiation of Ca^2+^ infusion illustrated a gradual loss of ΔΨm (i.e., increased TMRE fluorescence) over time in response to mitochondrial Ca^2+^ uptake (i.e., increased CaG5N fluorescence), respectively, measured until mPTP opening (Figure 1A,B). Overall, the 3 days typical traces of mitochondrial Ca^2+^ uptake illustrated an increase in CaG5N fluorescence, revealing a low Ca^2+^ buffering capacity and early mPTP opening between 5 and 35 min in the pTBI group compared to Sham (Figure 1B).

Quantitative analysis of loss of Ca^2+^ buffering capacity of pTBI revealed a “U-shaped” pattern across multiple post-injury time points in pTBI cohorts when compared to Sham (Figure 1C). Note that the Sham cohorts were conducted for each specific time point, and the data from each cohort were aggregated and illustrated as a single control histogram. Following injury, pTBI mitochondrial Ca^2+^ buffering capacity was moderately decreased at 30 min (29% decrease), 3 h (23% decrease), and 6 h (39% decrease) compared to Sham. A more robust decline in the loss of Ca^2+^ buffering capacity was noted at 24 h (76% decrease), 3 days (71% decrease), and 7 days (62% decrease) in pTBI vs. Sham. Later, at 14 days post-injury, there was a non-significant decrease (12% decrease) in the pTBI group vs. Sham.

### 3.2. Time Course of Decreased Mitochondrial Membrane Integrity Markers Post-pTBI

**Figure 2:**
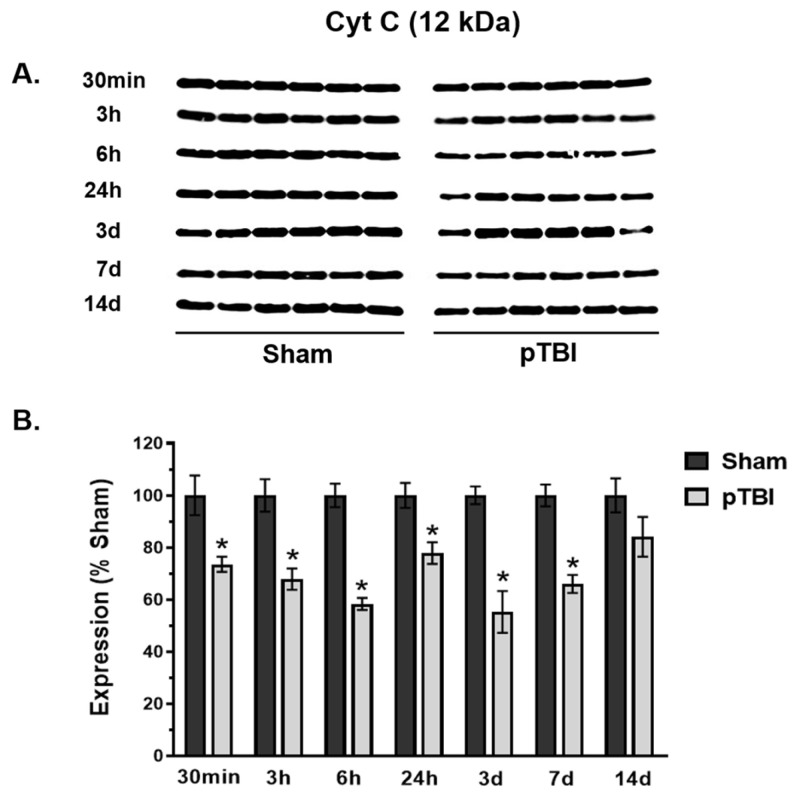
Time course analysis of Cyt C profile in mitochondria at the injury core post-pTBI. (**A**) Western blot images represent Cyt C time course in mitochondria expressed at 12 kDa. (**B**) Quantitative histogram results showed Cyt C decreased significantly starting at 30 min post-TBI (26% decrease) and remained significantly decreased for 3 h (32% decrease), 6 h (42% decrease), 24 h (22% decrease), 3 days (45% decrease), and at 7 days (34% decrease) post-pTBI compared to the respective Sham groups. Cyt C expression displayed a non-significant marginal decrease compared to Sham group levels at 14 days following pTBI. The values were presented as percentage change between the groups (Sham vs. pTBI). Bars represent group means  ±  SEM (N  =  6 animals per group). * *p*  <  0.05 compared to respective Sham control group (unpaired *t*-test).

**Figure 3:**
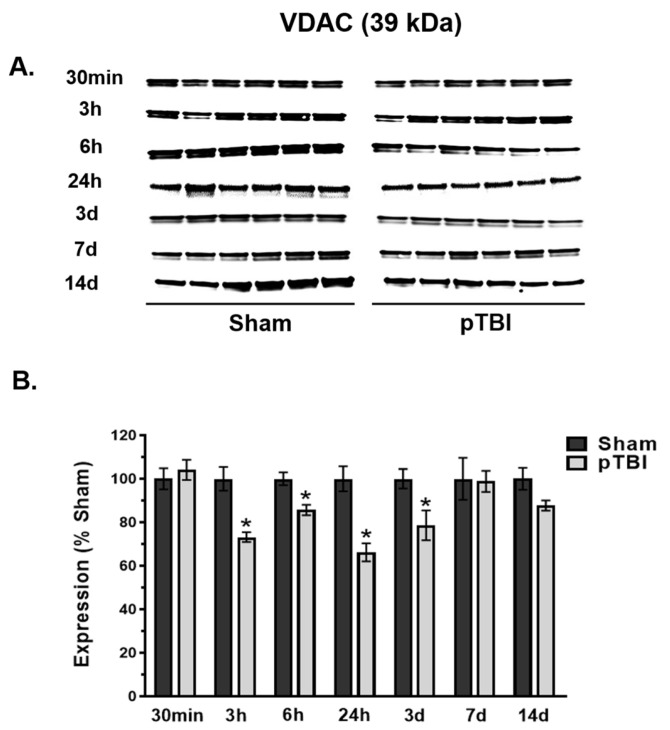
Time course analysis of VDAC profile in mitochondria at the injury core post-pTBI. (**A**) Western blot images represent VDAC time course in mitochondria, expressed at 39 kDa. (**B**) The quantitative histogram results for VDAC showed significant decreases at 3 h (27% decrease), 6 h (14% decrease), 24 h (34% decrease), and 3 days (22% decrease) post-pTBI compared to the Sham group. VDAC expression recovered to Sham level at 7 days and 14 days post-pTBI. The values are presented as percentage change between the groups (Sham vs. pTBI). Bars represent group means  ±  SEM (N  =  6 animals per group). * *p*  <  0.05 compared to the respective Sham control group (unpaired *t*-test).

We performed time courses of mitochondrial membrane proteins as membrane integrity markers following pTBI. The mitochondrial inner membrane protein Cyt C and outer membrane protein VDAC expression displayed “U-shaped” patterns at various time points quantified in pTBI up to 14 days post-injury compared with Sham (Figure 2 and Figure 3) using the Western blot total protein normalization method reported earlier [25,26].

The Cyt C protein band visualized at 12 kDa showed a marked decrease in the pTBI group vs. Sham (Figure 2A). More specifically, the Cyt C protein quantification revealed a significant decrease in its expression in pTBI cohorts, as soon as 30 min to 7 days post-injury (Figure 2B). The Cyt C content was significantly decreased by 23–45% at these acute post-injury time points, where 3 days (45% decreased) showed a maximum loss of membrane integrity protein Cyt C following pTBI. At 14 days, Cyt C showed a non-significant marginal decrease (16% decrease) in pTBI compared to Sham.

The mitochondrial outer membrane protein VDAC is considered a pivotal component of membrane integrity. It plays an important role in mPTP formation and is thought to be released from the outer membrane compartment upon insults causing membrane rupture due to mitochondrial swelling. The VDAC band was observed at 39 kDa (Figure 3A). Overall, the VDAC expression in the pTBI group showed a significant decrease between 3 h and 3 days (14–34% decrease) compared to Sham, where maximal loss of VDAC protein in mitochondria was observed at 24 h post-injury (Figure 3B). At 30 min, 7 days and 14 days post-injury, the VDAC expression remained identical to Sham.

Overall, the loss of inner and outer membrane mitochondrial proteins (e.g., Cyt C and VDAC) in pTBI compared to Sham control provided important evidence of loss of mitochondrial structural integrity markers after pTBI-induced secondary injury up to 7 days post-injury. The loss of mitochondrial membrane proteins could suggest their release from the mitochondrial membrane into the cytosol. This release may trigger apoptotic or necrotic cell death cascades in the aftermath of pTBI.

### 3.3. Temporal Changes in Mitochondrial Cell Death Markers Post-pTBI

**Figure 4:**
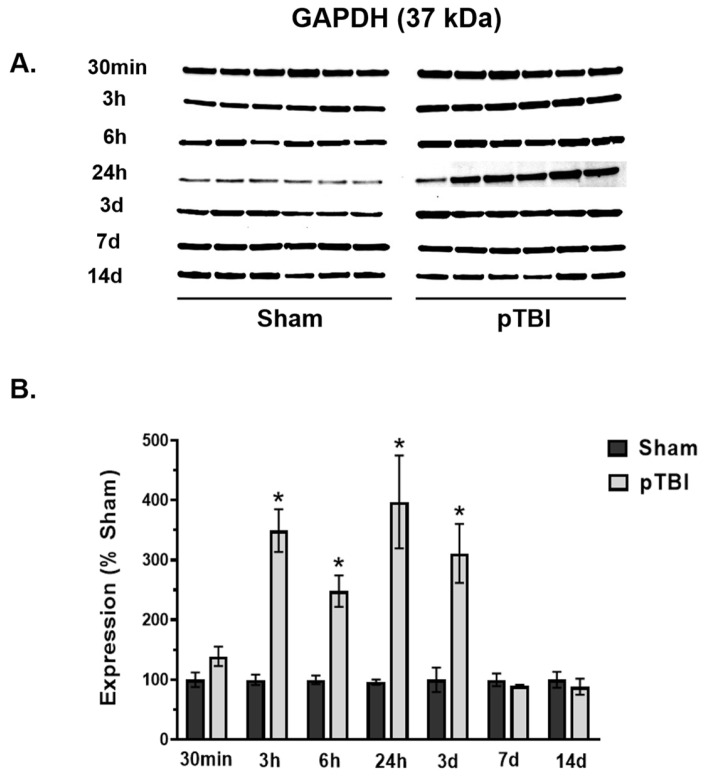
Time course analysis of GAPDH profile in the mitochondria at the injury core post-pTBI. (**A**) Western blot images represent GAPDH time course in the mitochondria, expressed at 37 kDa. (**B**) The quantitative histogram results for GAPDH showed significant increases at 3 h (249% increase), 6 h (148% increase), 24 h (297% increase), and 3 days (211% increase) post-pTBI. GAPDH decreased to Sham group levels at 7 days and 14 days following pTBI. The values are presented as percentage change between the groups (Sham vs. pTBI). Bars represent group means  ±  SEM (N  =  6 animals per group). * *p*  <  0.05 compared to the respective Sham control group (unpaired *t*-test).

**Figure 5:**
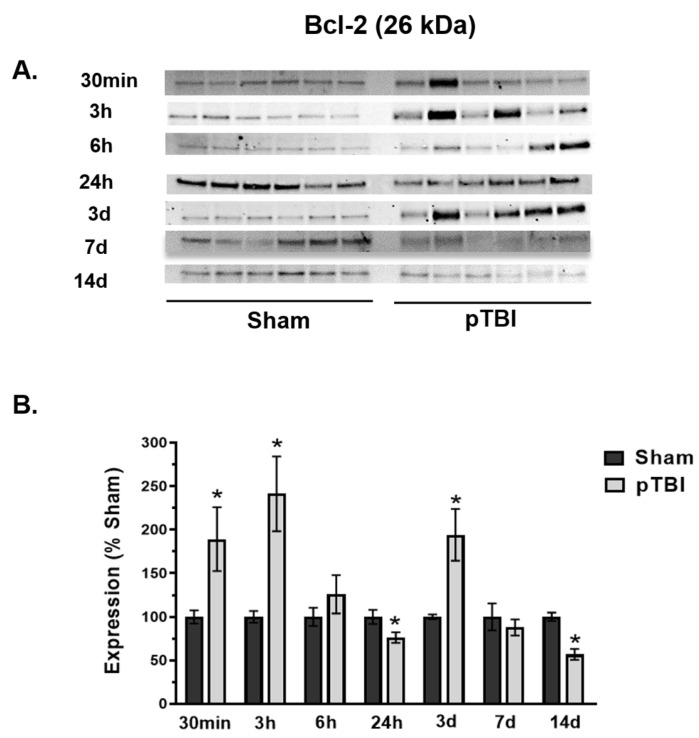
Time course analysis of Bcl-2 profile in mitochondria at the injury core post-pTBI. (**A**). Western blot images represent the time course of Bcl-2 in the mitochondria, expressed at 26 kDa. (**B**). The quantitative histogram results for Bcl-2 showed significant increase at 30 min (89% increase), 3 h (142% increase) and 3 days (94% increase) post-pTBI compared to Sham group. In contrast, Bcl-2 expression decreased significantly at 24 h (24% decrease) and 14 days (44% decrease) post-pTBI compared to Sham. Bcl-2 did not show any significant change at 6 h and 7 days after pTBI. The values were presented as percentage change between the groups (Sham vs. pTBI). Bars represent group means  ±  SEM (N  =  6 animals per group). * *p*  <  0.05 compared to the respective Sham control group (unpaired *t*-test).

We evaluated the down-stream effects of pTBI-induced cell death response markers at various time points. Here, we examined two potential markers that may offer preliminary insights into stimulated cell death-responsive markers following pTBI. We quantified mitochondrial GAPDH and Bcl-2 marker protein expression at various time points up to 14 days post-injury, which illustrated an “inverted U-shaped” injury response pattern in pTBI vs. Sham (Figure 4 and Figure 5).

The GAPDH protein expression was visualized at 37 kDa (Figure 4A), followed by quantification in the pTBI vs. Sham groups at each post-injury time point (Figure 4B). In pTBI, the GAPDH protein content showed a modest elevation (39% increase) at 30 min, which was not statistically significant. However, a significant elevation was observed between 3 h and 3 days post-injury, with increases ranging from 2.5 to 4 folds compared to the Sham group, with the peak elevation occurring at 24 h. At 7 days and 14 days post-injury, the GAPDH expression remained identical to Sham.

Bcl-2 expression was detected at 26 kDa (Figure 5A), followed by quantification in pTBI vs. Sham at each post-injury time point (Figure 5B). The Bcl-2 protein content was significantly elevated between 30 min (89% increase), 3 h (142% increase), and 3 days (94% increase) following pTBI compared to Sham. Bcl-2 expression remained unchanged at 6 h and 7 days post-injury. Bcl-2 expression significantly decreased at 24 h (24% decrease) and 14 days (43% decrease) in pTBI compared to Sham. Overall, the elevated response of both GAPDH and Bcl-2 proteins during acute time points suggested stimulated cell death regulatory responses following pTBI.

## 4. Discussion

Immediately following mechanical brain trauma, the initiation of excitotoxic responses plays a critical role in driving the progression of secondary injury and cell death. When addressing clinical TBI heterogeneity using pre-clinical TBI models and evaluating post-injury responses using several mild to severe pre-clinical models, it has been acknowledged that mitochondrial dysfunction is a common indicator of cellular damage and plays a pivotal role in post-injury excitotoxicity progression [3,4,5,6]. Key processes including mitochondrial ΔΨm generation, Ca^2+^ handling, mPTP, energy metabolism, and free radical generation play vital roles during the progression of TBI. These metabolic responses are intricately linked with various downstream pathological cascades that ultimately contribute to neuronal death. Several published reports have delved into pre-clinical TBI models and elucidated the cellular mechanisms at play after injury [7,8,9,10,11]. They underscore the critical importance of maintaining mitochondrial homeostasis as a central focus for mitigating the devastating impacts of TBI pathology [12,13,14]. Moreover, it is imperative to highlight that the temporal dynamics of mitochondrial-targeted excitotoxic indicators during the acute and sub-acute post-injury phase—spanning the vital first 2 weeks—remained inadequately explored in established pre-clinical TBI models. This gap in understanding presents an opportunity for further investigation, promising the potential to unveil new strategies for therapeutic intervention in TBI management.

The current study evaluated the temporal profile of mitochondrial excitotoxicity markers after severe pTBI, which may help us to understand mitochondria-mediated secondary injury pathology and aid in future screening of military-relevant therapeutics after TBI. Our findings expanded upon our earlier reports of metabolic pathway disruptions, impaired mitochondrial oxidative phosphorylation and underscoring dynamic changes in the oxidative profile within 30 min to 14 days post-pTBI [23,24,26,33].

**Figure 6:**
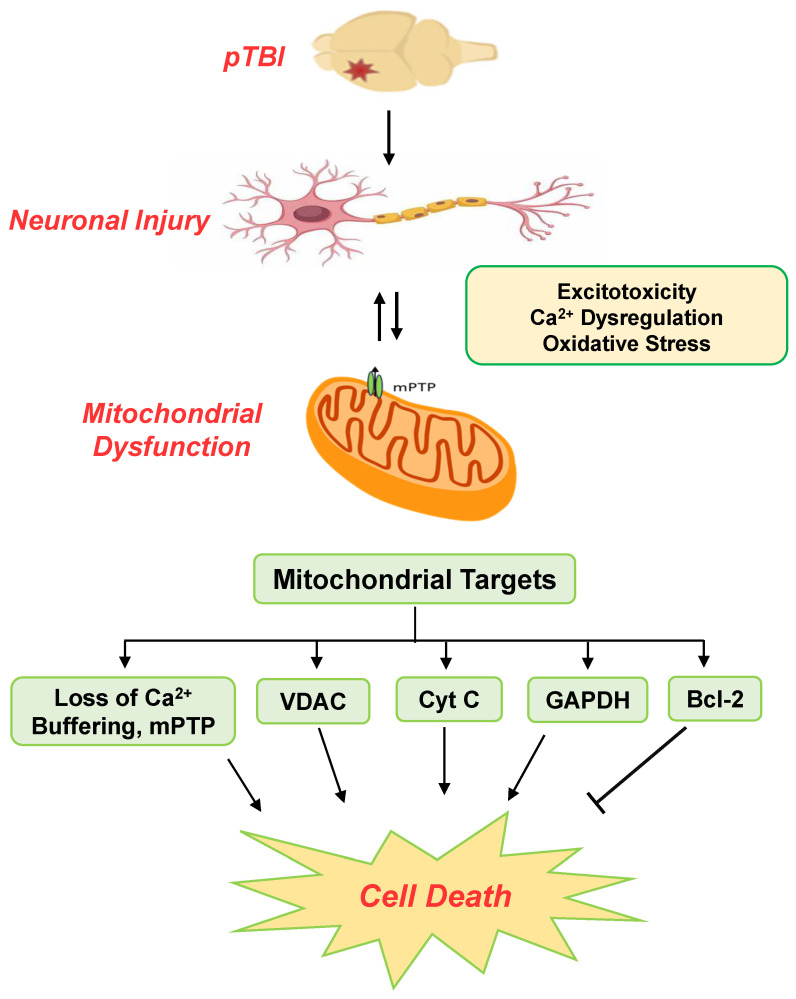
The major elements of mitochondria-centric excitotoxicity response following pTBI. Penetrating TBI occurs due to external mechanical assault to the brain, which leads to the initiation of downstream cascades of secondary injury mechanisms, including elevated excitotoxicity, oxidative stress, and Ca^2+^ dysregulation responses, over the post-injury period. These processes are deeply intertwined, creating a complex web of interdependence, intricated by mitochondrial dysfunction. As intracellular Ca^2+^ levels surge, a disruption of mitochondrial Ca^2+^ homeostasis followed by early mPTP and a dysregulation of protein targets such as Cyt C, VDAC, GAPDH, and Bcl-2 may further underscore downstream pathophysiological responses, leading to cell death after brain trauma. The temporal changes identified in such crucial mitochondrial targets may facilitate comprehensive evaluation of TBI therapeutics following neurotrauma.

Excitotoxicity driven by NMDA receptor overactivation is a primary contributor to these mitochondrial changes. This leads to an influx of intracellular Ca^2+^ to toxic levels [34,35,36]. Elevated intracellular Ca^2+^ is transferred into the mitochondrial matrix, driven by the electrochemical gradient established by ΔΨm [36]. A persistent elevation of mitochondrial Ca^2+^ further destabilizes Ca^2+^ homeostasis, particularly following repeated NMDA receptor activation [37]. Mitochondrial Ca^2+^ overload ultimately results in mPTP opening, triggering a cascade of events including apoptosis and neuronal death [34,35,36]. The intricate relationships between Ca^2+^ imbalance, mitochondrial function, and apoptotic signaling pathways following pTBI have been graphically represented (Figure 6).

Under normal physiological conditions, cytoplasmic Ca^2+^ concentrations are approximately 100 nM, but they can rise to 1 μM during stress or injury. Mitochondrial Ca^2+^ levels, by contrast, are roughly 10,000 times higher than cytoplasmic levels and play a critical role in maintaining cellular Ca^2+^ homeostasis [38,39]. During mild stress, increases in mitochondrial free Ca^2+^ (0–0.3 μM) enhance ATP production by activating the TCA cycle dehydrogenase enzymes [40]. However, sustained elevations in Ca^2+^ concentration up to 1 μM impair mitochondrial respiration, lead to mPTP opening, and result in the release of pro-apoptotic signals [41].

Dysregulation of intracellular Ca^2+^ homeostasis has been identified as a key driver of secondary injury mechanisms following TBI [42,43]. Temporal real-time ex vivo measurement of mitochondrial ΔΨm, Ca^2+^ buffering capacity, and mPTP opening are important parameters for understanding the progression of excitotoxic responses of pTBI over time. As observed earlier [25], a decrease in mitochondrial Ca^2+^ buffering capacity is an early indicator of mitochondrial dysfunction further supported by mitochondrial swelling and ATP synthesis failure. This cascade is closely regulated by the interplay between the mPTP, Cyt C, VDAC, and Bcl-2 family proteins and subsequent cell death [44,45].

In our rodent pTBI model, we observed significant time-dependent changes in mitochondrial Ca^2+^ buffering capacity, membrane integrity, and apoptotic response markers over a period of 14 days post-injury. These findings expanded upon earlier reports of metabolic pathway disruption and impaired mitochondrial oxidative phosphorylation and underscore the dynamic changes in the oxidative profile within 30 min to 14 days post-pTBI [23,24,26]. This study broadens the focus by evaluating mitochondrial dysfunction specifically focusing on Ca^2+^ dynamics and its critical role in cell death.

Our results revealed a progressive decline in Ca^2+^ buffering capacity from 30 min to 7 days post-pTBI, with the most significant reduction observed at 24 h (Figure 1C). Interestingly, the Ca^2+^ buffering capacity exhibited a “U-shaped” pattern with a partial improvement in Ca^2+^ buffering capacity at 14 days post-injury. The “U-shaped” pattern of Ca^2+^ buffering capacity is partially related to the biphasic nature of mitochondrial bioenergetics decline we observed earlier [24], where the first phase displayed moderate loss, and the second phase showed a more robust ATP synthesis failure in pTBI compared to Sham [24]. Notably, our Ca^2+^ buffering capacity data aligns closely with the bioenergetics parameters at 3 days post-pTBI, indicating that both metrics reached their peak depletion at 3 days time point [24]. While Ca^2+^ buffering capacity showed notable recovery at 14 days, mitochondrial bioenergetics remained compromised at this time point. These findings underline the interplay between Ca^2+^ buffering handling and bioenergetics responses during the secondary injury phase of pTBI and highlight the importance of targeting these processes for therapeutic intervention.

Impairment of Ca^2+^ buffering and early mPTP opening has been linked to many diseases including myocardial ischemia–reperfusion injury [46], hepatic ischemia–reperfusion injury [47], TBI [48,49], premature aging [50], and PD [51]. Furthermore, early mPTP opening in TBI initiates a self-amplifying vicious cycle of Ca^2+^ imbalance, ROS generation, mitochondrial swelling, and mitochondrial outer membrane permeabilization, resulting in severe cellular damage [52].

Under normal physiological conditions, the mPTP remains closed, preserving mitochondrial integrity. VDAC regulates the passage of small molecules and prevents the release of Cyt C into cytosol. Additionally, the membrane integrity markers Cyt C and VDAC are traditionally considered housekeeping proteins due to their essential roles in cellular metabolism and homeostasis. Interestingly, we noted that the decrease in Ca^2+^ buffering capacity is accompanied by a significant depletion in Cyt C and VDAC levels with a “U-shaped” pattern at early time points up to 7 days post-injury (Figure 2 and Figure 3).

Cyt C, located at the mitochondrial intermembrane space, plays a crucial role in transferring electrons between Complexes III and IV in the electron transport chain (ETC). The release of Cyt C into the cytosol is a critical step in initiating apoptosis, as it binds to apoptosomes and activates caspase cascades [53]. Our findings revealed a decrease in Cyt C content between 30 min and 7 days following pTBI (Figure 2B). This observation indirectly aligned with TBI CCI injury studies, where Cyt C translocated from the mitochondria to the cytosol, initiating DNA fragmentation and mitochondrial-dependent apoptosis [54,55].

Since the Cyt C protein is an essential component of the ETC, it is plausible that a disruption of mitochondrial bioenergetics and membrane integrity would accompany the release of Cyt C. Previous research has demonstrated that reduced expression of mitochondria specific manganese superoxide dismutase (MnSOD) enhances Cyt C release in a CCI model of TBI [54]. Similarly, in our earlier study, we observed a time-dependent decrease in MnSOD levels following a pattern similar to Cyt C reduction in the pTBI time course [25,26]. Once released into the cytosol, Cyt C activates caspase-3 in a time-dependent manner [56,57]. However, Cyt C translocation itself, along with caspase activation/inhibition events, may be sufficient to induce neuronal cell death [55]. Interestingly, both Cyt C and activated caspase-9 have been detected in the cerebrospinal fluid (CSF) of severe TBI patients, suggesting that extracellular Cyt C release could serve as a potential biomarker for injury severity [58]. Excitotoxicity, primarily mediated by NMDA receptor activation, exacerbates mitochondrial Ca^2+^ uptake, leading to increased membrane permeability, transient Cyt C release, and elevated ROS production, all promoting apoptosis [59,60,61]. During apoptosis, Cyt C release from mitochondria is critically regulated by the VDAC, further amplifying apoptosis mediated cell signaling and neuronal cell death.

The outer mitochondrial membrane protein VDAC act as a gatekeeper for ions and metabolites and regulates mitochondrial function and cellular homeostasis. As a key component of forming the mPTP, VDAC play a central role in apoptosis by mediating the release of Cyt C and other pro-apoptotic signaling factors into cytosol. We observed a significant alteration of VDAC levels starting between 3 h and 3 days post-pTBI (Figure 3B). VDAC is critical for maintaining ΔΨm by facilitating the flux of ATP, ADP, and Pi, which are essential for bioenergetics. Structural changes in VDAC, such as oxidation, may contribute to mitochondrial outer membrane permeabilization and impaired Ca^2+^ homeostasis. VDAC oxidation observed following TBI leads to conformational changes that compromise mitochondrial membrane integrity and increase susceptibility to swelling and rupture of the outer mitochondrial membrane [62]. This ultimately exacerbates apoptosis and neuronal damage.

Subsequently, we observed that the cell death regulation markers GAPDH and Bcl-2 proteins spiked in an “inverted U-shaped” pattern after pTBI (Figure 4 and Figure 5). GAPDH, traditionally known as a housekeeping gene and a stable reference for gene expression studies, showed a time-dependent increase between 3 h and 3 days post-injury (Figure 4). Emerging data suggest that GAPDH plays diverse roles beyond glycolysis, acting as an intracellular sensor and a modulator of cellular homeostasis or apoptosis in response to stress. Post-translational modifications such as acetylation, phosphorylation, S-nitrosylation, and aggregation enable GAPDH to sense intra- and extracellular stress and determine cellular fate [63]. Under stress conditions, GAPDH translocates to the mitochondria, where it contributes to apoptosis by inducing mitochondrial outer membrane permeabilization (MOMP), releasing Cyt C and AIF and disrupting ΔΨm [63,64,65,66]. However, GAPDH can also participate in cellular recovery, facilitate DNA repair, and regulate redox balance via transcriptional regulators [63,67,68,69,70]. This dual role of GAPDH suggests that GAPDH elevation in mitochondria following pTBI may be contributing as a regulator of apoptotic mediated cell death or survival pathways. However, more rigorous studies are required to illustrate this in more detail.

Furthermore, GAPDH was found to be significantly oxidized following TBI [62]. GAPDH aggregation has been linked to mitochondrial dysfunction and neuronal death in AD [71] and TBI [72]. This aggregation has been shown to trigger necrotic cell death via mPTP opening, which was prevented by the small molecule RX624, a hydrocortisone derivative, leading to improved motor function in TBI rats [73,74]. These findings suggested that the post-injury GAPDH aggregation may exacerbate mitochondrial dysfunction, highlighting the need for further investigation into its role in apoptosis and the potential for therapeutic targeting.

Bcl-2 levels also revealed an “inverted U-shaped” pattern at different time points post-pTBI compared to Sham animals (Figure 5B). Early post-injury time points of Bcl-2 expression in pTBI showed a noticeable increase at the 30 min, 3 h, and 3 days time points, whereas Bcl-2 content remained unchanged/decreased in other time points. Bcl-2, a key regulator of apoptosis located on the outer mitochondrial membrane, promotes cell survival by inhibiting pro-apoptotic proteins like Bax and Bak. Additionally, it regulates intracellular Ca^2+^ homeostasis, acting as a channel regulator to control apoptotic signaling [75,76,77,78]. Bcl-2 family proteins directly interact with VDAC, preventing Cyt C release and mPTP opening [78,79], thereby maintaining membrane permeability [80]. At clinical levels, an upregulation of Bcl-2 has been documented in pericontusional brain tissue and CSF following acute neurotrauma [80], thus underscoring its potential as a biomarker following neurotrauma. The time-dependent changes in Bcl-2 observed in our study suggested an elevation of compensatory mechanisms as the injury progresses over time. Nevertheless, the biphasic pattern of elevated Bcl-2 response is noticeable with a 2-fold upregulation during this acute post-injury period and is worth exploring as a possible early pTBI biomarker.

Overall, mitochondrial Ca^2+^ levels significantly modulate ΔΨm, bioenergetics, downstream cell signaling, and cell death/survival pathways, but their interplay in TBI remains poorly understood [81]. During excitotoxic insults, excessive Ca^2+^ uptake leads to ΔΨm collapse, ROS bursts, and reduced ATP synthesis [5,82,83,84,85,86,87]. The opening of mPTP serves as a critical checkpoint, triggering mitochondrial dysfunction and amplifying apoptosis signaling. Our findings highlighted the synergy between altered Ca^2+^ dynamics, membrane integrity markers, and apoptosis pathways post-pTBI. Understanding the temporal profiles of markers after a pTBI is essential for developing effective treatments to reduce further damage. Our study highlights critical times for intervention to address post-TBI pathology. Immediate strategies should focus on regulating Ca^2+^ levels in mitochondria and preventing the opening of the mPTP. Therapeutic options in an acute phase of neurotrauma may include increasing calcium-binding proteins like calretinin and calbindin-D28K and targeting pathways such as mTORC1-SKN-1-Nrf to maintain Ca^2+^ balance [88,89]. Promising targets also include sodium–calcium exchangers (NCXs) and blockers like pyrimidine-2,4,6-triones [90] and mPTP inhibitors like cyclosporin A and NIM-811 [49].

Maintaining mitochondrial function early on can prevent cell death and may lead to better outcomes post-pTBI. Thus, targeting changes in Ca^2+^ buffering and signals that promote cell death is vital. The sustained alterations in Ca^2+^ buffering, combined with the continued depletion of Cyt C and VDAC and the peaking “inverted U-shaped” responses of GAPDH and Bcl-2, signify both a prolonged phase of mitochondrial compromise and ongoing apoptotic signaling. Strategies aimed at preventing Cyt C interaction with cardiolipin (CL) via inhibiting Cyt C/CL peroxidase complexes and suppression of CL peroxidation are identified as prime targets for anti-apoptotic drug development [91]. Initiating drugs that can decrease neuronal apoptosis within 2 weeks post-pTBI may potentially limit secondary neuronal damage during the sub-acute phase. Indeed, administration of anti-apoptotic compounds like flavopiridol, oscovitine, and olomoucine has shown to improve lesion volume and prevent cognitive deficits in rodent models of TBI [92].

Overall, the first two weeks after pTBI represent a therapeutic window, with innate recovery mechanisms observed by 14 days, which was also noted in our prior study on the temporal profiling of oxidative stress [26]. Key recovery processes may include mitophagy, which removes damaged mitochondria, and mitochondrial biogenesis, which creates new ones [40,93]. Neuroplasticity, the brain’s ability to rewire itself, is also vital [94]. Additionally, there is potential for healthy mitochondria to be transferred from astrocytes to injured neurons, supporting energy restoration [95].

In summary, our study sheds light on the important role of temporal dynamics in mitochondrial excitotoxicity markers following severe TBI. We uncovered an intricate relationship involving calcium dysregulation, compromised mitochondrial membrane integrity, and apoptotic signaling pathways. Excitingly, we found time-dependent changes in calcium buffering capacity, as well as unique patterns in Cyt C and VDAC levels and shifts in GAPDH and Bcl-2. This underscores the critical need for timely therapeutic strategies that focus on mitochondrial calcium balance and mPTP opening. Our findings pave the way for innovative targeted therapeutics aimed at preserving mitochondrial function, ultimately helping to minimize secondary injuries and neuronal cell death after TBI. Additionally, pursuing long-term studies beyond 14 days is vital for a deeper comprehension of the secondary pathology related to TBI.

## Data Availability

The original contributions presented in this study are included in the article. Further inquiries can be directed to the corresponding author.

## Abbreviations

TBI: traumatic brain injury; pTBI: penetrating traumatic brain injury; ΔΨm: Mitochondrial Membrane Potential; Bcl-2: B-Cell Lymphoma 2; GAPDH: Glyceraldehyde 3-Phosphate Dehydrogenase; Cyt C: Cytochrome C; mPTP: Mitochondrial Permeability Transition Pore; VDAC: voltage-dependent anion channel; CaG5N: Calcium Green-5N; TMRE: Tetramethyl Rhodamine Ethyl Ester; Ca^2+^: calcium.
